# Multi Targeted CAR-T Cell Therapies for B-Cell Malignancies

**DOI:** 10.3389/fonc.2019.00146

**Published:** 2019-03-12

**Authors:** Nirav N. Shah, Theresa Maatman, Parameswaran Hari, Bryon Johnson

**Affiliations:** ^1^Division of Hematology and Oncology, Medical College of Wisconsin, Milwaukee, WI, United States; ^2^Department of Medicine, Medical College of Wisconsin, Milwaukee, WI, United States

**Keywords:** CAR-T, antigen escape, B-cell NHL, B-cell ALL, immunotherapy

## Abstract

Chimeric antigen receptor (CAR) modified T cell therapy has revolutionized the treatment of relapsed and refractory hematological malignancies. Through targeting of the CD19 antigen on B cells durable remissions have been achieved in patients with B cell non-Hodgkin lymphoma and acute lymphoblastic lymphoma. Despite impressive responses, multiple escape mechanisms to evade CAR-T cell therapy have been identified, among which the most common is loss of the target antigen. In this review we will highlight outcomes to date with CD19 CAR-T cell therapy, describe the current limitations of single targeted CAR-T therapies, review identified tumor escape mechanisms, and lastly discuss novel strategies to overcome resistance via multi-targeted CAR-T cells.

## Introduction

Adoptive cell transfer utilizing autologous T cells genetically engineered *ex vivo* to target tumor antigens has revolutionized the treatment of relapsed, refractory hematological malignancies. T cells can be engineered to express a new T cell receptor (TCR) or a chimeric antigen receptor (CAR) to target tumor-associated antigens. CAR-modified T-cells are composed of a single-chain variable fragment (scFv) that binds tumor antigens and is fused to a spacer and transmembrane domain with intracellular costimulatory signaling domains, most commonly CD28 or 4-1BB with CD3ζ (1, 2). While multiple tumor antigens are under active clinical investigation, CAR-T cell therapy against the CD19 receptor on B cells is most clinically advanced. CD19 is a 95kDa glycoprotein present on the B cell surface from early development until differentiation into plasma cells. Its normal function involves regulation of signal transduction through the B cell receptor. CD19 was an ideal first target as its expression is restricted to B lineage cells and it is not found on pluripotent blood stem cells or on most other normal tissues (3). These anti-CD19 CAR-T (CAR-19-T) cells have demonstrated significant efficacy in the treatments of patients with relapsed, refractory B cell lymphoid malignancies (4–7). Their potential was first highlighted in a series of case reports that demonstrated the potential of CD19 targeting in patients with non-Hodgkin lymphoma (NHL) (8, 9). Since these initial few reports, the field of CAR-T cell therapy has exploded and now data is available from several large multi-center studies reporting clinical outcomes from Phase II trials (4, 6, 10). Although these studies demonstrated unprecedented efficacy, it also became apparent that not all patients respond to CAR-19-T cells, and even for those who initially respond, durability of response remains a limitation. Amongst the earliest identified resistance mechanisms was the downregulation of target antigen CD19 from tumor cell surface (11, 12).

To date three Phase II studies have reported on efficacy data in B cell NHL and B cell acute lymphoblastic lymphoma (ALL). First, in NHL, Neelapu et al. reported their results of ZUMA-1, a Phase II study of CD28 CD3ζ CAR-19-T cells for relapsed, refractory large B cell lymphoma. Among 108 patients treated and followed for a minimum of 1 year, 42% of patients remained in response at the time of publication. In a subset of patients who relapsed and had available data, CD19-negative relapse was observed as the likely mechanism of failure (6). The JULIET study evaluated the efficacy of a 41BB CD3ζ CAR-19-T cell as part of an international, phase 2 clinical trial. Among 93 treated patients, the 3 month CR rate was 32% (13). This identical construct was concurrently explored in a similar international phase II study for pediatric and young adult patients with relapsed, refractory B cell ALL. Following treatment, the 3 month overall response rate was 81% with 59% of these patients remaining alive and relapse-free at 12 months. Among relapsed patients, the majority (15/22) presented with CD19-negative disease, demonstrating a major limitation of currently FDA-approved CAR-T therapies. For patients with CD19-negative relapse, options are limited with few approved therapies (14), and prognosis is generally poor although there is great promise with a number of clinical trials underway targeting alternative B-cell antigens such as CD22 (15). In this review we will focus on the role of target antigen loss as a mechanism of CAR-T failure and strategies for overcoming this current limitation through novel CAR constructs.

## Antigen Loss as a Major Limitation of CAR-T Cell Therapies for B Cell Malignancies

While initial response rates in patients treated with CAR-T cells for B cell malignancies have been impressive when compared to historical outcomes for patients with relapsed, refractory disease, many patients fail to respond, and others relapse after initially responding. Of the known escape mechanisms, the best defined etiology of disease relapse has been due to target antigen loss, and recent clinical data indicated that 7–33% of responders in CAR-19-T cell trials for B-ALL have relapsed due to loss of cell-surface CD19 (12, 16), which supports the immunoediting hypothesis proposed by Schreiber and colleagues in 2002 (17). CD19 loss after CAR-T therapy was recognized early on when one of two B-ALL patients relapsed 2 months after treatment with CAR-T cells following an initial complete response (11). Deep sequencing identified that the malignant CD19-negative clone was actually present in peripheral blood and marrow at day 23, a time when the patient was initially felt to not have residual disease (11).

**Figure 1:**
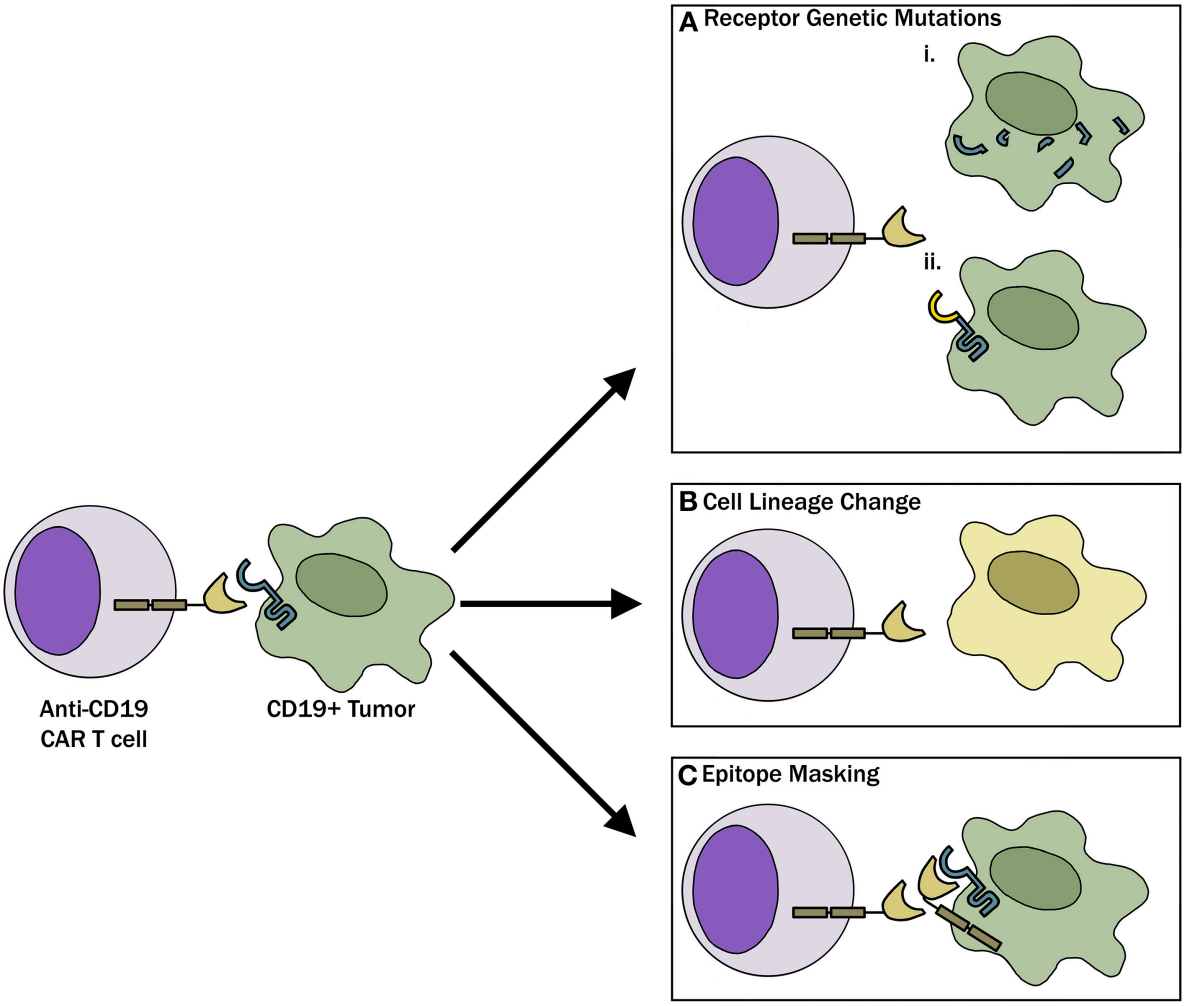
Mechanisms of CAR-T evasion. **(A)** Tumor cells, through genetic mutations, can either (i) completely lose CD19 receptor expression or (ii) modify the CD19 receptor such that CAR-T cells can no longer recognize and bind the target. **(B)** Tumor cells can undergo phenotypic switch to a different lineage that is inherently CD19 negative to evade CAR-T cells. **(C)** As described in the case report by Ruella et al. (18) lentiviral modification of a single leukemic cell allowed for epitope masking and evasion of CAR-T cell therapy.

With the recognition that antigen loss is a major barrier to CAR-T therapies, research has uncovered that there are multiple mechanisms responsible for the antigen loss (Figure 1). Following CAR-19-T cell treatment, Sotillo et al. identified both acquired mutations and alternatively spliced CD19 alleles in the malignant B cells of pediatric patients with relapsed disease (19). This resulted in either no cell surface CD19 expression or surface of expression of CD19 variants that no longer contained the epitope recognized by the CAR-T cells. A study by Fischer et al. suggested that CD19 isoforms lacking the CAR-T binding epitope are present in some patients prior to treatment, predisposing these individuals to treatment failures (20). These observations have been questioned in a more recent study where antigen loss in a cohort of 12 B-ALL patients was found to be due to a variety of loss of heterozygosity mutations, and alternative splicing only occurred with rare frequency (21). Bagashev et al. identified retention of mutated, misfolded CD19 proteins in the endoplasmic reticulum, suggesting another possible mechanism responsible for antigen loss (22).

Another mechanism involved in antigen loss after CAR-T cell therapy is cell lineage switch. One of the first observations regarding lineage switch was reported in 2015 by Evans and colleagues, where a CLL patient with Richter transformation relapsed after CAR-19-T cell treatment with a plasmablastic lymphoma which is inherently CD19 negative (23). This finding has been followed up by a report showing that 2 of 7 patients with mixed lineage leukemia (MLL)-rearranged B-ALL relapsing with CD19-negative AML following treatment with CD19 CAR-T cells (24) and a recent case report where a pediatric patient with TCF3-ZNF384 fusion-positive B-ALL had a myeloid switch after therapy (25). In an intriguing recent report, Ruella et al. described a novel mechanism of CD19 evasion. This patient with CD19-negative relapse was identified to have a single CD19-positive leukemic cell transformed during the CAR-T manufacturing process (18). The investigators showed that CD19 CAR on the leukemia surface bound in cis to CD19, thereby masking it from being recognized by the CAR (18). Although this is likely an extremely rare event, it represents a not previously described mechanism of resistance and highlights the importance of having rigorous manufacturing standards in place when engineering T cells for adoptive immunotherapies.

Partial antigen loss due to antigen down-regulation, in contrast to complete loss of antigen, has also been implicated as a mechanism for resistance to CAR-T cell therapy (15, 16, 26–28). Using a CD20 CAR, Murata and colleagues were the first to document that a threshold level of around 200 antigen molecules per target cell were required to induce lytic function, while approximately 10-fold higher numbers of molecules were needed to stimulate cytokine production (26). Another study documented that a CD30 CAR could selectively target lymphoma cells while “ignoring” CD30+ hematopoietic progenitor cells (HPCs) due to differential levels of antigen expression (27). The low levels of CD30 on HPCs were insufficient to trigger significant cytolysis, unlike the high levels that were present on the lymphoma cells. Mackall et al. showed that not only is CAR-T cell function regulated by target antigen density on malignant cells, but also by CAR density on the engineered T cells (28). More recently, this same laboratory documented that relapses in patients treated with a CD22 CAR directly correlated with diminished levels of CD22 on the B-ALL cells (15). The investigators went further to show in animal studies that differential levels of CD22 on leukemia cells could have a dramatic impact on anti-cancer efficacy. These results have future implications not only for the use of CAR-T therapy in hematologic malignancies, but also as the use of CAR-T cells for solid tumors moves forward.

While the body of evidence for antigen loss in B cell leukemias after CAR-T therapy is indisputable, the role for antigen loss in similarly treated lymphoma patients has been more challenging since immunohistochemistry has typically been used to assess antigen levels rather than flow cytometry. Suggesting the role of antigen loss in lymphoma is the report by Shalabi et al. that documented sequential loss of CD19 and CD22 antigens in a patient with DLBCL following CAR T cell therapies that targeted these proteins (29). It is clear that more sophisticated ways of assessing antigen loss after CAR T cell treatment of lymphoma patients will be required to determine just how frequently this occurs.

## Targeting Multiple Molecules to Overcome the Limitation of Antigen Loss in CAR-T Cell Therapies

**Figure 2:**
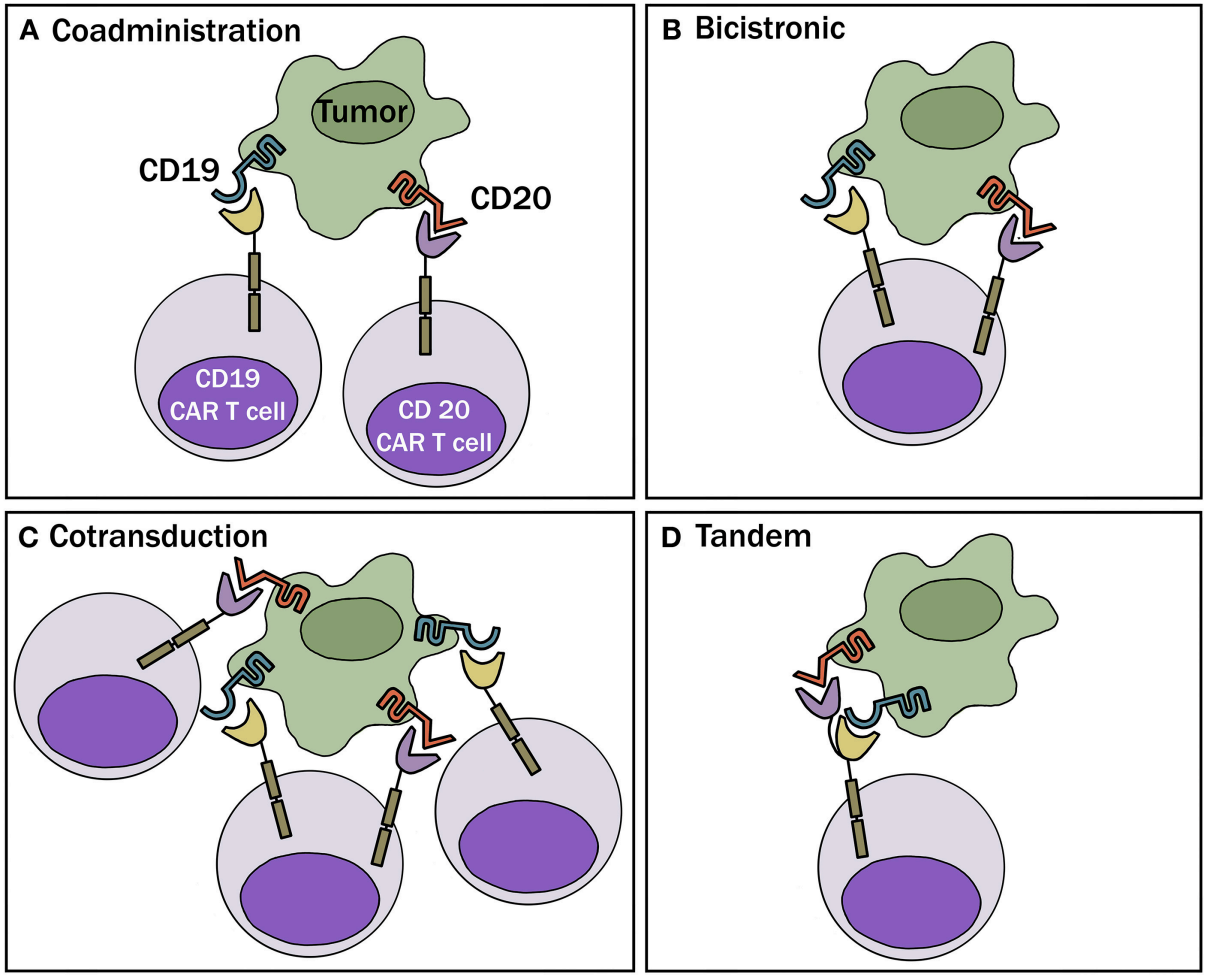
Multi-targeted CAR-T approaches. **(A)** Coadministration—involves production of two separate CAR-T cell products infused together or sequentially. **(B)** Bicistronic vector—allows expression of 2 different CARs on the same cell. **(C)** Cotransduction—encode 2 CAR constructs via transduction with multiple vectors. With this process, one will also obtain cells that express each CAR alone. **(D)** Tandem—encode 2 CARs on same chimeric protein using a single vector.

One obvious way to combat the problem of antigen loss following CAR-T cell therapy is by targeting more than one antigen receptor. This can be accomplished by 1 of 4 different approaches: (a) Generate 2 or more cell populations expressing different CARs and infuse them together or sequentially (coadministration); (b) Use a bicistronic vector that encodes 2 different CARs on the same cell; (c) Simultaneously engineer T cells with 2 different CAR constructs (cotransduction), which will generate three CAR-T subsets consisting of dual and single CAR-expressing cells; or (d) Encode 2 CARs on the same chimeric protein using a single vector (i.e., bi-specific or tandem CARs) (Figure 2). These different approaches are highlighted in a recent review article by Majzner and Mackall (16).

One of the first pre-clinical studies that advocated for the use of more than one CAR to prevent emergence of antigen escape was in glioblastoma (30). In this study, T cells were either separately engineered to express HER2- or IL-13Rα2-specific CAR and mixed, or sequentially transduced to co-express the two constructs (approaches a & c above). Both approaches helped prevent antigen escape and provided better anti-tumor efficacy (30). Pre-clinical data in support of using dual-targeting in B cell malignancies emerged soon thereafter. In one of the first publications documenting successful use of a tandem CAR pre-clinically, Zah et al. developed a CD19-CD20 CAR and showed that the dual construct could prevent the spontaneous development of CD19-negative tumor cell variants in immune deficient mice (31). Later in 2016, Gill and colleagues tested 3 of the 4 approaches noted above (approaches a, b & c) where they simultaneously targeted CD19 and CD123 (IL-3 receptor α chain) (32). Using a xenograft mouse model, the investigators demonstrated that mixtures of CD19 and CD123 CAR-T cells or cells engineered to express both receptors on the same T cell, through co-transduction with separate lentiviral vectors or a bicistronic vector, could prevent antigen escape.

Preclinical results with another CD19–CD20 tandem CAR (approach d) were published by Schneider et al. (33). Constructs were generated where CD19 or CD20 was expressed as the distal receptor on the CAR protein (designated as CAR 1920 or CAR 2019, respectively) and compared to single antigen CARs. Both CAR 1920 and CAR 2019 tandem constructs were superior to CD19 single-CAR in a murine xenograft leukemia model. CD19 expression on Raji leukemia cells (express both CD19 & CD20) was strongly diminished by coincubation with CD19 single CAR-T cells but maintained at higher levels by coincubation with CAR 1920 or CAR 2019 T cells. Interestingly, when CAR-T cells were stimulated with antigen-positive leukemia cells, expression of the CD19/CD20 tandem constructs resulted in less cytokine production than CD20 CAR alone, suggesting some attenuation of signaling when the CD19 and CD20 receptors were co-expressed in tandem on the same CAR. Finally, in a high-burden mouse leukemia model, CAR 2019 T cells provided improved anti-leukemia efficacy over that induced by single CD19 or CD20 CAR-T cells or mixtures of CD19 and CD20 single-expressing CAR T cells (33). There did not appear to be a clear advantage of expressing the CD20 receptor distal or proximal to the CD19 receptor, but the CAR 2019 did show better binding of CD20 peptide and improved killing against some cancer cell lines *in vitro* (33). These preclinical findings were translated into a Phase 1, first-in-human bispecific CAR-T cell trial with an anti-CD19/anti-CD20 tandem receptor (NCT03019055). Early results from this dose-escalation study demonstrated an ongoing complete response (CR) or partial response (PR) in 3/6 heavily pre-treated and relapsed B cell NHL patients treated with CAR-20.19-T cells. Interestingly among the three patients who progressed or relapsed, all retained either CD19 or CD20 positivity on subsequent biopsy suggesting other mechanisms rather than antigen loss as the etiology of therapy failure (34).

Similar to the development of a CD20.CD19 CAR-T cell, Fry and colleagues developed a bispecific CD19-22 CAR (15). The CD19-22 CAR was able to efficiently kill CD19+ and CD22+ human leukemia target cells *in vitro*, secrete IFN-γ in response to the target cells, and eradicate the leukemia in immune deficient mice. A Phase 1 clinical trial is currently underway testing this construct in patients with relapsed, refractory Diffuse Large B-cell Lymphoma and B-cell ALL. Early results from this dose escalation trial has demonstrated 2 patients with a CR among seven treated patients (35).

As a result of the encouraging preclinical data, several tandem CARs and combined or sequential administration of single CARs are being tested in the clinic (Table 1). Table 1 also includes an ongoing clinical trial that uses an “armored” CAR, which encodes a CD19 receptor, CD3 and CD28 signaling motifs, the costimulatory ligand 4-1BBL, as well as a suicide gene safety system if the cells mediate severe acute toxicities. Although this vector does not target more than one antigen receptor, the idea is that the armored CAR-T cells might be able to prevent antigen escape by providing a more vigorous initial response that would eliminate the malignant cells before antigen escape develops.

**Table 1:** Actively recruiting ClinicalTrials.gov registered studies using tandem CARs or administration of multiple single CARs.

**CAR**	**NCT number**	**B cell malignancy**	**Site**
Sequential CD19, CD20	NCT03207178	Non-specified	Shanghai, China
Multiple mixtures (CD19 + CD22, CD38, CD20, CD123, CD70, or CD30)	NCT03125577	Non-specified	Guangzhou, Shenzhen & Kunming, China
“Armored” CD19	NCT03085173	CLL	New York, NY, USA
CD19–CD20 dual	NCT03398967	Leukemia, Lymphoma	Beijing, China
	NCT03019055	Lymphoma, CLL	Milwaukee, WI, USA
CD19–CD22 dual	NCT03614858	Leukemia	Suzhou, China
	NCT03593109	Lymphoma	Xi'an, China
	NCT03468153	Lymphoma	Shanghai, China
	NCT03448393	Leukemia, Lymphoma	Bethesda, MD, USA
	NCT03398967	Leukemia, Lymphoma	Beijing, China
	NCT03330691	Leukemia, Lymphoma	Seattle, WA, USA
	NCT03289455	Leukemia	London & Manchester, UK
	NCT03287817	Lymphoma	London, Manchester & Newcastle, UK
	NCT03241940	Leukemia	Palo Alto, CA, USA
	NCT03233854	Lymphoma	Palo Alto, CA, USA

## Other Multi-Targeting Approaches for Hematologic Malignancies Involving CARs

One interesting approach that evolved from work done by Vie and colleagues (36) is the engineering of T cells to express CD16 (FcγRIII) CARs so that they are capable of mediating antibody-dependent cellular cytotoxicity (ADCC). The first of these CARs contained CD16 linked to intracytoplasmic domains of FcγRIII (36). More recently, CD16 CARs have been created by adding CD3ζ and CD28 or 4-1BB signaling domains (37–39). Basically, one can administer engineered CD16 CAR-T cells along with one or more of the several tumor antigen-specific monoclonal antibodies that are known to facilitate ADCC [reviewed in Caratelli et al. (40)]. This is an attractive approach because it could allow for the targeting of multiple antigens simultaneously, as long as each of the monoclonal antibodies facilitates ADCC. Two clinical trials using this approach to treat patients with B cell malignancies in conjunction with anti-CD20 (rituximab) are currently recruiting patients (NCT02776813, NCT02315118).

Other armored CARs in development include an IL-18-secreting CD19 or MUC16 CAR, which appears to modulate the tumor microenvironment of both hematologic malignancies and solid tumors and helps enhance endogenous anti-tumor T cell responses (41). CARs with the same specificities have also been modified to co-express a PD-1 blocking moiety or to secrete IL-12 (42). Interestingly, local PD-1 blockade at tumor sites could increase anti-tumor activity of the CAR T cells while avoiding the toxicities associated with systemic PD-1 blockade (42). The IL-12-secreting MUC16 CAR was able to modify the tumor microenvironment by deleting tumor-associated macrophages and enhancing CAR T cell proliferation and cytotoxicity (43).

Finally, it is notable that the development of trivalent CARs has now been reported (44, 45). It will be interesting to see if these, and other current and future multi-targeting CAR approaches, are able to obviate the problem of antigen loss. Only time will tell.

## Limitations of Multi Targeted CAR-T approaches

While potential advantages of multi-targeted CAR-T approaches over the current standard of care have been discussed, there are several unanswered questions regarding safety, efficacy, and feasibility of these products. First, multi-targeted CAR-Ts do not address other proposed resistance mechanisms outside of target antigen loss. Recently, Fraietta et al. reported on the determinants of efficacy and resistance of CD19 CAR-T cells in CLL (46). They demonstrated that the intrinsic transcriptome profile of the CAR-T cells determined efficacy with CAR-T cells enriched in memory-related genes and IL-6/STAT3 signaling seen in responding patients, while upregulation of genes involved in T-cell differentiation and exhaustion were found in non-responding patients (46). Other proposed mechanisms include inhibition of CAR-T cells due to engagement of PD-L1 on tumor cells (42). In both scenarios, it is unlikely that multi-targeting would be able to overcome these resistance mechanisms. Second, there is limited understanding on the safety profile and *in vivo* activity of multi-targeted CAR-T cells in patients. It is possible that multi-targeting, through availability of increased target antigen, may lead to a more robust form of CRS, making their administration prohibitive. It is also unclear if the cytotoxic activity that is seen *in vivo* is due to preferential engagement of one target over the other, or in the setting where more than one CAR-T cell product is co-administered, whether there will be equal engraftment and distribution of the modified cells. Lastly, a significant concern of multi-targeting is the cost associated with production. Several approaches to multi-targeted CAR-T cells requires >1 viral transduction or >1 manufacturing run, which when commercialized, can significantly increase the cost of therapies that are already exceedingly expensive.

## Conclusions

CD19 CAR-T cell treatments have transformed the management of B cell hematological malignancies. Despite the remarkable outcomes in relapsed, refractory patients, soon after its development the presence of resistance mechanisms was identified, and CD19-negative relapse was the dominant pathology described. Loss of CD19 has occurred through a variety of mechanisms including genetic modification, leading to partial or complete down regulation of the CD19 receptor, or truncation of the protein preventing binding by CD19 CAR-T cells (16). Other mechanisms include lineage switching and the development of a phenotype that is intrinsically CD19-negative (23, 24). Finally, it was most recently reported that through viral transfection of a CAR in a single leukemic cell, the patient developed a CD19 resistant leukemic clone that resulted in patient death (18). Regardless of the mechanism, it is apparent that single targeting of CD19 leads to selective pressure and development of tumor cell clones that can evade CD19 CAR-T therapy. It is possible that multi-targeted CAR-T cell therapy may overcome this resistance mechanism and improve clinical outcomes. Many trials are now in development or actively accruing patients to determine if targeting multiple antigens can prevent treatment failure due to CD19 loss and improve response rates and durability of response. Pending results of these studies, FDA approved CAR-19-T cell products will remain the mainstay treatment option for relapsed, refractory B cell NHL and ALL.

## Author Contributions

NS contributed to development, writing, and final review of the article. TM contributed to illustrations and final review of the article. PH contributed to development and final review of the article. BJ contributed to development, writing, and final review of the article.

### Conflict of Interest Statement

NS, PH, and BJ have research funding from Lentigen Technology. NS has participated in advisory boards for Juno and Kite pharmaceuticals. The remaining author declares that the research was conducted in the absence of any commercial or financial relationships that could be construed as a potential conflict of interest.
